# Symbiotic nitrogen fixation and endophytic bacterial community structure in *Bt*-transgenic chickpea (*Cicer arietinum* L)

**DOI:** 10.1038/s41598-020-62199-1

**Published:** 2020-03-25

**Authors:** Das Alok, Harika Annapragada, Shilpa Singh, Senthilkumar Murugesan, Narendra Pratap Singh

**Affiliations:** 1 Division of Plant Biotechnology, Indian Institute of Pulses Research, Kalyanpur, Kanpur India; 2 Division of Basic Sciences, Indian Institute of Pulses Research, Kalyanpur, Kanpur India

**Keywords:** Rhizobial symbiosis, Microbiome

## Abstract

Symbiotic nitrogen fixation (SNF) of transgenic grain legumes might be influenced either by the site of transgene integration into the host genome or due to constitutive expression of transgenes and antibiotic-resistant marker genes. The present investigation confirmed proper nodulation of five tested *Bt*-chickpea events (IPCa2, IPCa4, IPCT3, IPCT10, and IPCT13) by native *Mesorhizobium* under field environment. Quantitative variations for nodulation traits among *Bt*-chickpea were determined and IPCT3 was found superior for nodule number and nodule biomass. Diversity, as well as richness indices, confirmed the changes in bacterial community structure of root and root-nodules from *Bt*-chickpea events IPCa2 and IPCT10. Especially, Gram-positive bacteria belonging to *Firmicutes* and *Actinobacteria* were selectively eliminated from root colonization of IPCa2. Richness indices (CHAO1 and ACE) of the root-associated bacterial community of IPCa2 was 13–14 times lesser than that of parent *cv* DCP92-3. Root nodule associated bacterial community of IPCT10 was unique with high diversity and richness, similar to the roots of non-*Bt* and *Bt*-chickpea. It indicated that the root nodules of IPCT10 might have lost their peculiar characteristics and recorded poor colonization of *Mesorhizobium* with a low relative abundance of 0.27. The impact of *Bt*-transgene on bacterial community structure and nodulation traits should be analyzed across the years and locations to understand and stabilize symbiotic efficiency for ecosystem sustainability.

## Introduction

Chickpea (*Cicer arietinum L*.) is a self-pollinating diploid (2n = 16) grain legume with a genome size of 738 Mb, grown and consumed all over the world, especially in the Afro-Asian countries^1^. As the major chickpea producing country, India contributes 61.49% of global production with the productivity of 0.951 t/ha^2^. However, chickpea productivity is stagnating (<1000 kg/ha) due to several biotic and abiotic stresses. Gram pod borer (*Helicoverpa armigera* Hubner) is the major devastating pest which attack chickpea starting from first fortnight after sowing, voraciously during flower and pod development stages resulting in yield loss of up to 20–30% annually. Due to the successful introduction of *Bt*-cotton in India, the pest sequently moves from cotton to other crops including pigeonpea and chickpea. Polyphagous, high fecundity and insecticide resistant nature of pod borer led to the damage to pods ranging from 30–95% with 400 kg/ha yield losses^3,4^. Conventional breeding approaches for insect resistance have limited success in chickpea due to lack of sufficient resistance source and incompatibility with many of the wild relatives^5^. Success of *Bt*-cotton in India led extensive research on developing transgenic crops especially, insect resistance *Bt*-chickpea^6–12^. Site of insertion of transgene in plant genome is critical as it could alter plant metabolism and toxicity of *Bt*-protein^13–16^. Similarly, insertion of the transgene in active coding region of genome might be responsible for hemizygous state of some *Bt*-chickpea events with distorted segregation ratio^12^. Altered plant metabolism of genetically modified (GM) plants along with constitutive expression of chimeric *Bt*-transgenes and neomycin phospho-transferase II (*npt*II) can also influence the diversity, and richness of endophytic bacterial community that are often described as an important modulating agent of plants’ fitness to adapt against environmental stresses.

The endophytic bacterial community of transgenic-maize TC1507 (*Cry1F* and herbicide resistance phosphinothricin-N-acetyltransferase) differed from that of MON810 (*Cry1Ab*) and their near-isogenic parent. Highest species diversity (Shannon) and richness (Chao 1) were reported in TC1507 and MON810, respectively, but the values were not statistically significant^17^. Changes in the organic carbon content in the rhizosphere of non-*Bt* and *Bt*-brinjal can be attributed to alterations in the quality and composition of root exudates that could further be responsible for fluctuations in density and diversity of actinomycetes population.

Especially in grain legumes, effect of transgenics on symbiotic nitrogen fixation that fertilizes crop and soil should be assessed. Transgenic trait impaired several BNF parameters in glyphosate-resistant (GR) or ROUNDUP READY RR-soybean, with transgene coding 5-enolpiruvylshikimic acid-3-phosphate synthase-EPSPS^18^. Nodule dry weight (NDW) was the attribute most negatively affected by the transgenic trait, with reductions ranging from 10 to 21% and congruent with the decrease in percent total nitrogen as ureides (%NU) in the transgenic cultivars^19^. Despite the lack of yield penalty in transgenic legumes, their long term impact on symbiotic efficiency and rhizosphere ecosystem should be monitored. Hence, present investigation is aimed to understand the possible changes in nodulation and bacterial community structure associated with root and nodule tissues of five *Bt*-chickpea lines *vis a vis* parent control *cv* DCP 92-3.

## Results

### Effect of Bt-transgene integration on nodulation of transgenic chickpea

Field experiment using surface sterilized seeds of non-*Bt* and *Bt*-chickpea confirmed proper germination and nodule initiation process. However, nodule number and their fresh biomass varied among the transgenic events (Table 1). IPCT 3 recorded the lowest nodule number at early vegetative stage, and recovered at later stages and recorded highest nodule number at pod initiation stage along with IPCT 4. IPCa 4 recorded the lower nodule biomass compared to parent *cv* DCP 92-3 while IPTC 3 recorded the highest nodule biomass. This particular transgenic event improved the nitrogen availability in the rhizosphere to 2.47 g N/kg soil (Table 2). However, the maximum nitrogen content of shoots at early vegetative stage is reported with IPCa 2 and IPCT 10 followed by IPCa 4. N-content of shoots declined in transgenic events compared to parent during later stages of plant growth except for IPCa 4.

**Table 1:** Nodulation traits of *Bt*-transgenic chickpea.

Events	Nodule Number (Unit/plant)			Nodule Fresh Biomass (g/plant)		
	63DAS	75DAS	96DAS	63DAS	75DAS	96DAS
Parent *cv*. DCP 92-3	18 ± 2.5	24 ± 1.7	11 ± 2.2	1.61 ± 0.18	3.85 ± 0.32	8.62 ± 0.62
IPCa 2	19 ± 2.2	23 ± 2.5	18 ± 2.5	1.67 ± 0.17	4.85 ± 0.21	8.41 ± 0.50
IPCa 4	16 ± 2.2	24 ± 1.3	34 ± 3.1	1.28 ± 0.26	3.21 ± 0.27	7.54 ± 0.44
IPCT 3	12 ± 1.7	21 ± 2.5	32 ± 2.5	1.21 ± 0.20	4.44 ± 0.36	9.77 ± 0.67
IPCT 10	18 ± 2.9	27 ± 2.9	18 ± 1.7	1.49 ± 0.18	3.41 ± 0.19	6.12 ± 0.20
IPCT 13	16 ± 2.1	33 ± 2.6	20 ± 2.1	1.48 ± 0.21	4.29 ± 0.34	6.92 ± 0.50
CD (P = 0.05)	2.81	5.02	5.18	NA	0.70	1.09

**Table 2:** Yield and nitrogen content in the rhizosphere soil and plant tissues of *Bt*-chickpea.

Transgenic Events	N-content of rhizosphere soil (g/kg soil)	N content of chickpea root (%)	N-content of chickpea shoot (%)		Average grain yield (g/plant)*
			Early Stage	Late Stage	
Parent *cv*. DCP 92-3	1.77 ± 0.13	1.12 ± 0.12	1.77 ± 0.12	2.26 ± 0.07	17.57 ± 2.07
IPCa 2	1.25 ± 0.15	0.77 ± 0.11	2.17 ± 0.15	1.61 ± 0.15	13.30 ± 4.40
IPCa 4	1.10 ± 0.16	0.84 ± 0.12	2.15 ± 0.18	2.20 ± 0.14	15.67 ± 0.05
IPCT 3	2.47 ± 0.13	0.91 ± 0.15	1.87 ± 0.18	1.76 ± 0.10	14.23 ± 0.97
IPCT 10	0.60 ± 0.08	1.05 ± 0.14	2.17 ± 0.19	1.68 ± 0.15	15.73 ± 2.40
IPCT 13	1.90 ± 0.16	0.77 ± 0.12	1.54 ± 0.15	1.60 ± 0.07	15.40 ± 1.35
CD (P = 0.05)	0.289	NA	0.385	0.291	NA

*Average value of three replications in which grain yield of 30 plants per replication was considered.

### Taxonomic characterization of chickpea associated bacterial community

A total of 1,033,280 bacterial (V3-V4 of 16S rDNA) sequencing reads were obtained from root samples of chickpea with counts per sample ranging from 113,671 to 248,340. Sequencing of root-nodule samples resulted in a total of 606,112 sequence reads ranging from 58,237 to 155,545 per sample. Rarefaction curve for observed OTUs in root sample of IPCa2 tended to an asymptote and this indicated the sufficiency of sequence depth (Fig. S1). On the contrary, rarefaction curves of other samples did not saturate and thus OUT richness might be higher than the observed. Among the tested root-nodule samples except IPCT 10, rarefaction curves tended to asymptote. Rarefaction curves for Shannon and Simpson diversity indices reached the saturation plateau within approximately 10,000 sequence reads in every root and nodule samples.

**Figure 1:**
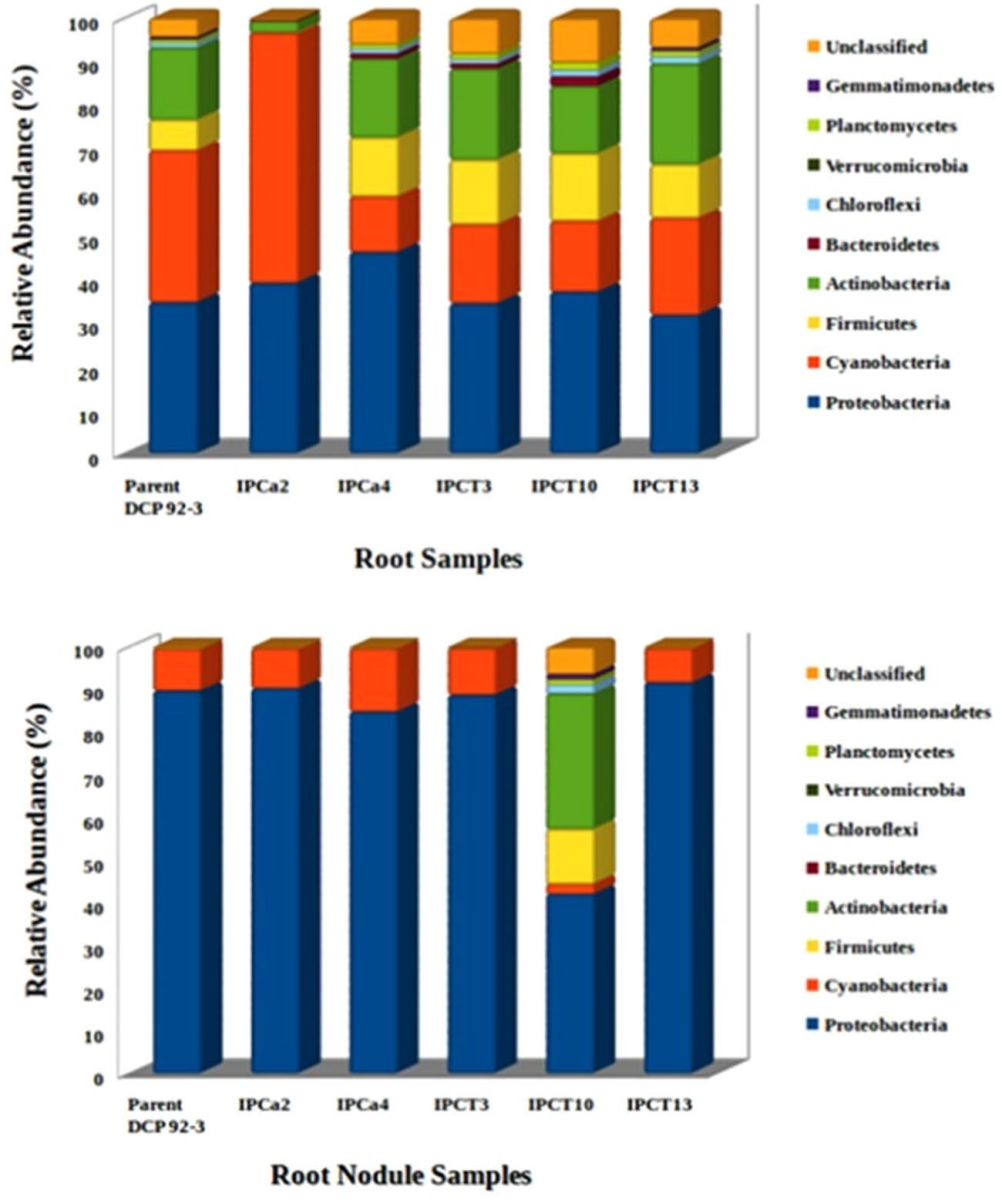
Relative abundance of root and root-nodule associated endophytic bacterial phyla of non-*Bt* and *Bt*-transgenic chickpea. Phylum level bacterial composition in root tissues of IPCa2 and root-nodules of IPCT10 are unique in comparison to rest of the *Bt*-chickpea and parent cultivar.

Read were classified taxonomically using a high performance version of the Ribosomal Database Project (RDP) Naïve Bayes taxonomic classification algorithm. Bacterial reads unclassified at phylum level is ranging from 0.3 to 10.06% with maximum in roots of transgenic event IPCT 10. Taxonomic classification of OTUs resulted in detection of 22–29 phyla in root and 15–28 phyla in root-nodule microbiome of chickpea (Table S1). However, only 7 phyla colonizing all tested root tissues and 2 phyla from root-nodules are considered as dominant phyla with relative abundance more than 0.01. Four major phyla detected in all tested chickpea tissue samples are *Proteobacteria, Cyanobacteria, Firmicutes*, and *Actinobacteria*. Phylum *Bacteroidetes* is detected in 75% of tested tissue samples. Roots of parent *cv* DCP 92-3 is colonized equally by *Proteobacteria* and *Cyanobacteria* with relative abundance of ~0.35. Other dominant bacterial phyla colonizing chickpea roots are *Actinobacteria* (0.17) > *Firmicutes* (0.07%) > *Chloroflexi* (0.009) > *Planctomycetes* (0.008) > *Gemmatimonadetes* (0.006). Bacterial colonization of transgenic event IPCT10 significantly differs from parent as well as other transgenic events (Fig. 1). Relative abundance of proteobacteria in nodule tissues of IPCT10 was 0.42 while that of root-nodule tissues from remaining transgenic lines is 0.85–0.92. This is followed by *Cyanobacteria* with relative abundance ranging from 0.08–0.15. *Firmicutes* and *Actinobacteria* are considered as rare phylum in most of the tested nodule tissues with relative abundance of <0.001.

Percentage of classified bacterial reads at Genus level is dropped to 34–92% in root tissues. Genus level taxonomical classification resulted in the detection of 311–655 genera in root tissues and 161–567 Genera in root-nodules. Prominent Genera associated with root tissues are *Calothrix, Rickettsia, Mesorhizobium, Methylobacillus, Arthrobacter, Bacillus, Streptomyces, Saccharopolyspora, Rhodococcus, Ramlibacter, Propionivibrio, Janthinobacterium, Kaistobacter, Sphingomonas, Ammoniphilus*, and *Rubrobacter* with relative abundance >0.01. *Mesorhizobium* is the most dominant bacteria in root -nodules with relative abundance ranging between 0.74–0.82. However, IPCT10 reported poor colonization of *Mesorhizobium* with relative abundance of 0.27. *Actinocatenispora, Pseudaminobacter, Burkholderia*, and *Shinella* are detected only in root-nodules with RA of >0.001.

### α-diversity of bacterial community associated with tissues of non-*Bt* and *Bt* transgenic chickpea

Number of bacterial species detected in each root sample ranges from 1230–1529 in root tissues except IPCa 2 that reported only 425 species (Table 3). Transgenic event IPCa 4 recorded maximum number of bacterial species. Similarly, bacterial species ranging from 144–197 were detected in root-nodules except one transgenic event IPCT 10 where 1196 bacterial species are detected. Root-nodules of chickpea cv. DCP 92-3 are colonized by four different species of *Mesorhizobium* i.e. *M. huakuii, M. septentrionale, M. camelthorni*, and *M. opportunistum* that collectively contribute 11.13% reads of *Mesorhizobium*. Remaining 88.87% reads of *Mesorhizobium* are not classified at species level. Interestingly, root-nodules of IPCa2 supported the colonization of *M. amorphae* that contributed 29.76% of total *Mesorhizobium* reads along with other species like *M. septentrionale, M. camelthorni*, and *M. huakuii. M. amorphae* was detected only in root -nodules of IPCa2. It was observed that the total number of V3-V4 reads from root-nodules of IPCT3 and IPCT10 i.e. 152814 and 155545 respectively are significantly higher than that of remaining samples (58237–92813) and the difference reflected on abundance of *Mesorhizobium* reads. However, the relative abundance (%) of *Mesorhizobium* in IPCT3 is similar to that of parent cultivar DCP 92-3. Transgenic event IPCT10 reported very low colonization of *Mesorhizobium* with 27.05% of total bacterial reads, and detected the presence of single species of *M. huakuii* that contribute 3.25% of 42067 *Mesorhizobium* reads (Table S2).

**Table 3:** Data Summary on metagenomic sequencing of v3-v4 regions of 16S rDNA.

Sample ID	No. of OTUs	No. of Phylum detected	No. of Genera detected	No. of Species detected	Richness Indices		Diversity Indices	
					CHAO	ACE	Shannon	Simpson
Root- Parent *cv*. DCP 92-3	17666	28	595	1302	32873.4	38045.2	5.9	0.8
Root- IPCa 2	1268	22	311	426	2538.4	2663.9	2.1	0.6
Root- IPCa 4	22198	29	655	1530	33547.3	38433.7	9.5	1.0
Root- IPCT 3	13609	26	587	1231	28201.1	32088.5	8.6	0.9
Root- IPCT 10	14934	28	622	1365	27206.2	31150.8	9.1	0.9
Root- IPCT 13	17555	29	617	1381	34772.6	40299.1	7.7	0.9
Nodule-Parent *cv*. DCP 92-3	426	15	192	198	999.4	1174.5	2.2	0.6
Nodule-IPCa 2	359	17	161	149	785.5	932.6	2.5	0.7
Nodule-IPCa 4	320	16	170	145	987.9	896.1	1.4	0.4
Nodule-IPCT 3	623	18	239	260	1185.4	1380.4	2.3	0.7
Nodule-IPCT 10	16456	28	567	1197	30196.5	34727.2	8.7	0.9
Nodule-IPCT 13	449	17	187	185	1179.1	1319.8	2.4	0.7

**Figure 2:**
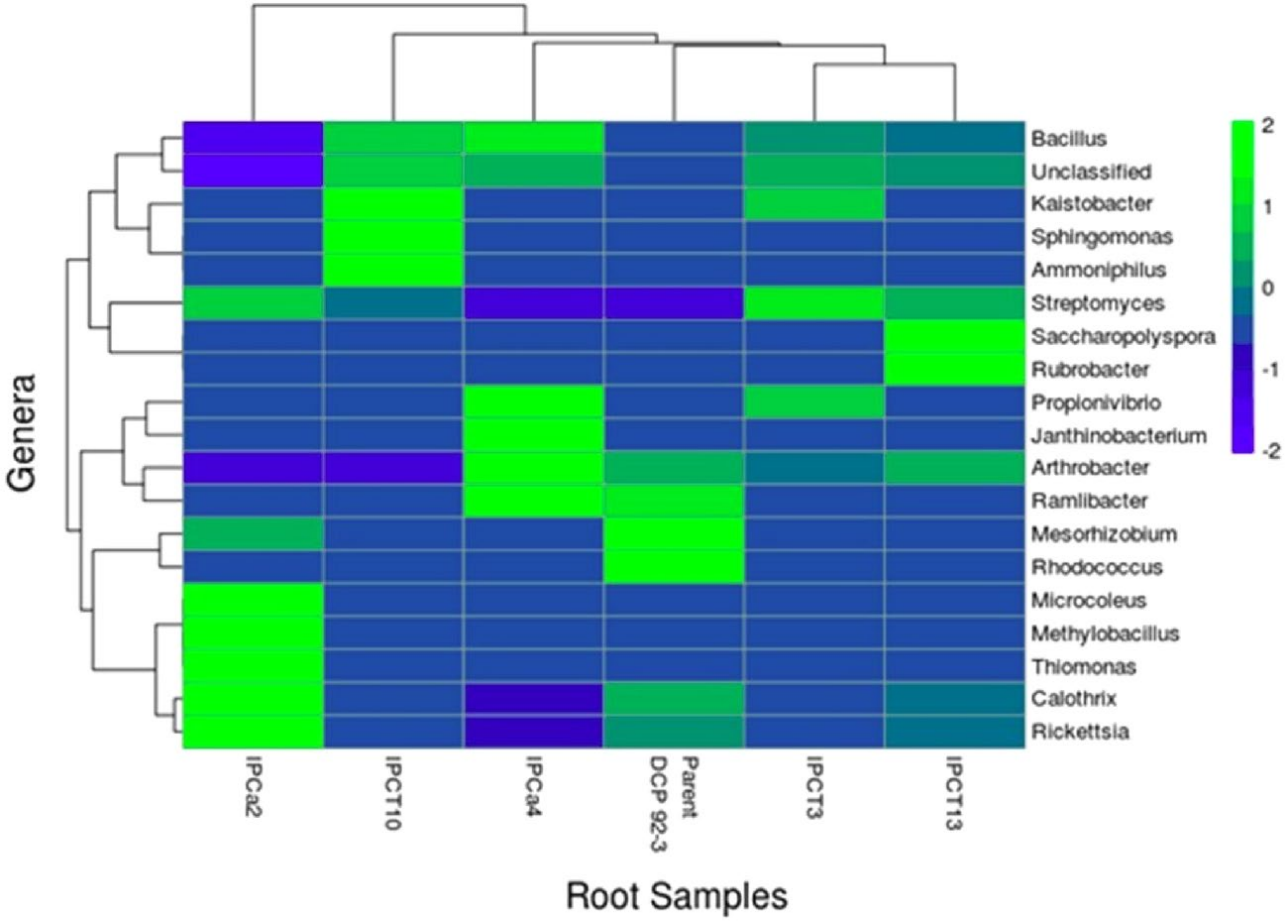
Heat map depicting root bacterial community structure of parent and transgenic chickpea.

**Figure 3:**
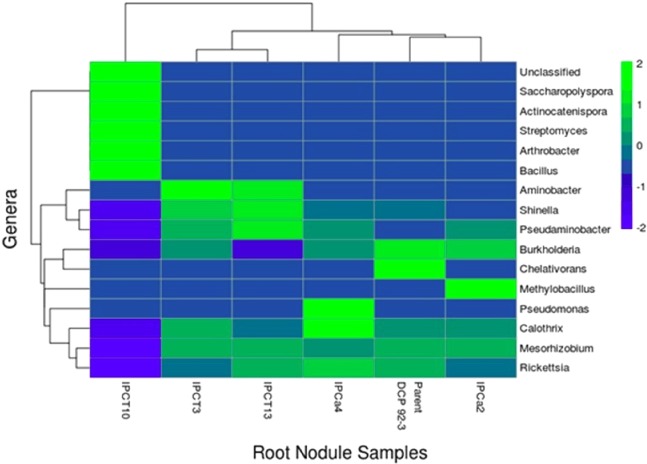
Heat map depicting root-nodule bacterial community structure of Non-*Bt* and *Bt*-chickpea. Root-nodule endophytic bacterial community structure of IPCT10 is unique and poorly colonized by *Mesorhizobium*.

**Figure 4:**
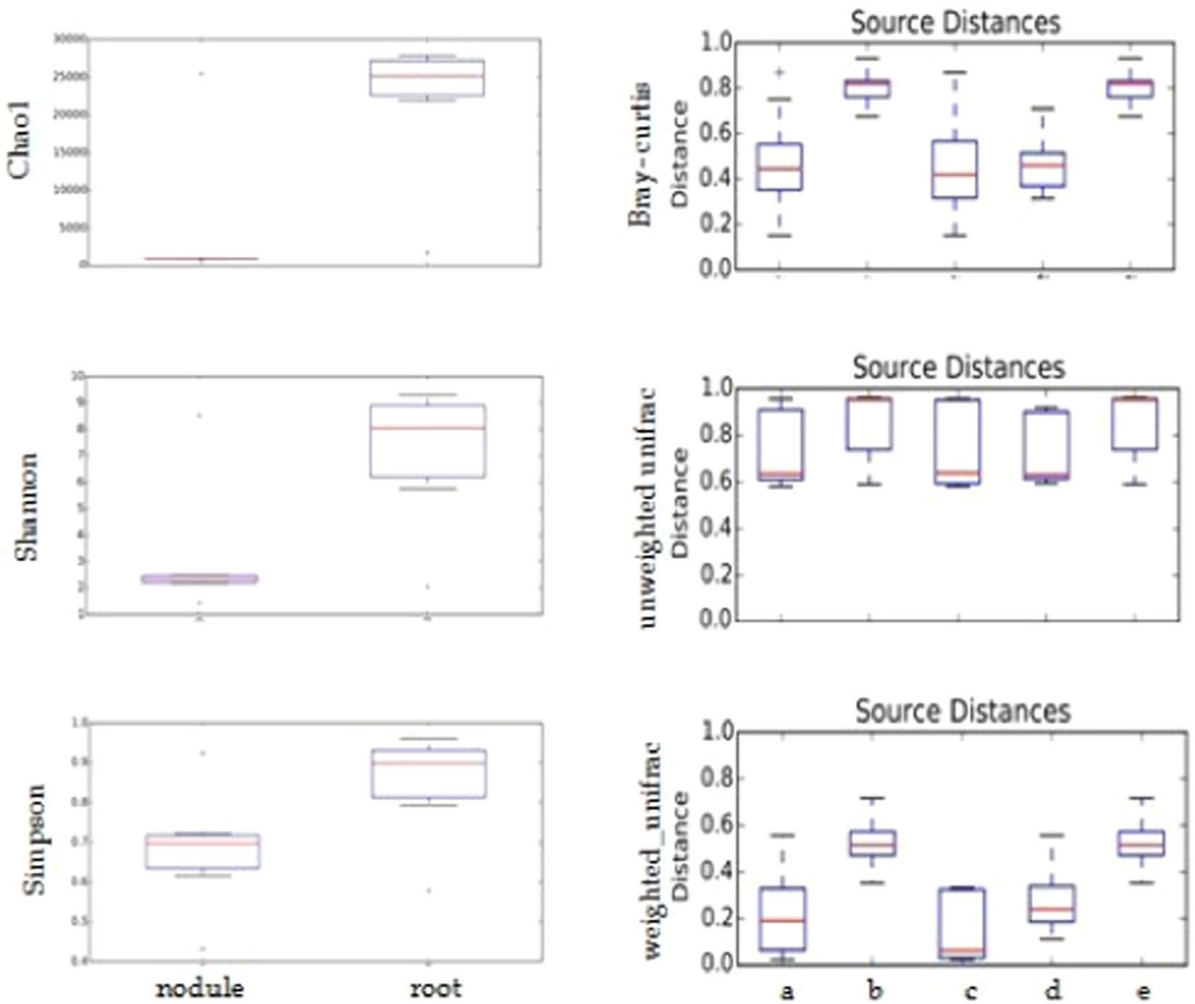
Box plot showing the differences in alpha diversity indices (Chao1, Shannon, and Simpson) and beta diversity indices (weighed and un-weighed UniFrac as well as Bray-Curtis) of bacterial community associated with plant chickpea tissues. Beta distance was calculated for the data set (**a**) all within sample sources, (**b**) all between sources, (**c**) nodule *vs* nodule (**d**) root *vs* root, (**e**) nodule *vs* root.

The heatmap revealed a significant difference of relative abundance of different Genus across the samples. It showed an obvious decrease in root-nodule colonization of IPCT 10 by Genera such as *Shinella, Pseudaminobacter, Burkholderia, Calothrix, Mesorhizobium* and *Rickettsia* compared to rest of the chickpea lines. Comparatively, higher abundance of Genus *Saccharopolyspora, Actinocatenispora, Streptomyces, Arthrobacter* and *Bacillus* was reported with root nodules of IPCT 10 (Fig. 2). In contrast, root tissues of IPCa 2 reported with poor colonization with *Bacillus* and *Arthrobacter*. It is the single event where the abundance of *Microcoleus, Methylobacillus, Thiomonas, Calothrix* and *Rickettsia* was increased in comparison to all other genotypes (Fig. 3). Box-Plot analysis revealed the lower bacterial diversity indices for Chao1, Shannon, and Simpson (p value < 0.05) in root-nodules compared to root tissues (Fig. 4).

### β-diversity of bacterial community associated with non-*Bt* and *Bt* transgenic chickpea

**Figure 5:**
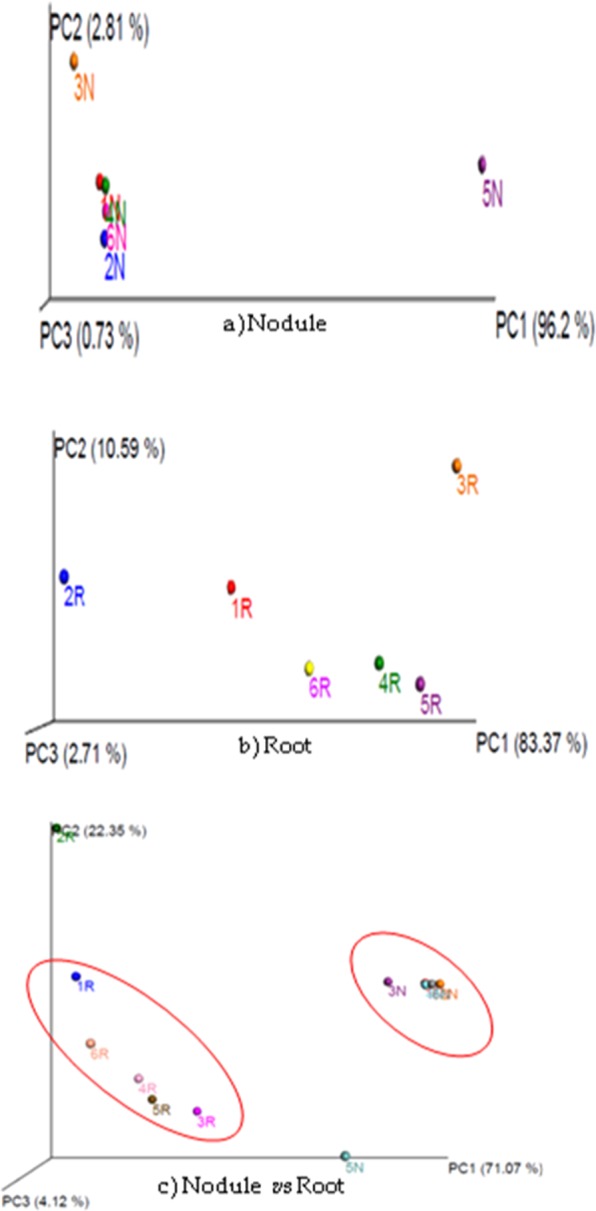
Principal Coordinate Analysis (PCoA) plot of samples using the weighted UniFrac distance metric: (**a**) nodule, (**b**) root, (**c**) nodule *vs* root. Note: Sample ID mentioned as 1N-6N and 1R -6R are as detailed in Table S1.

**Figure 6:**
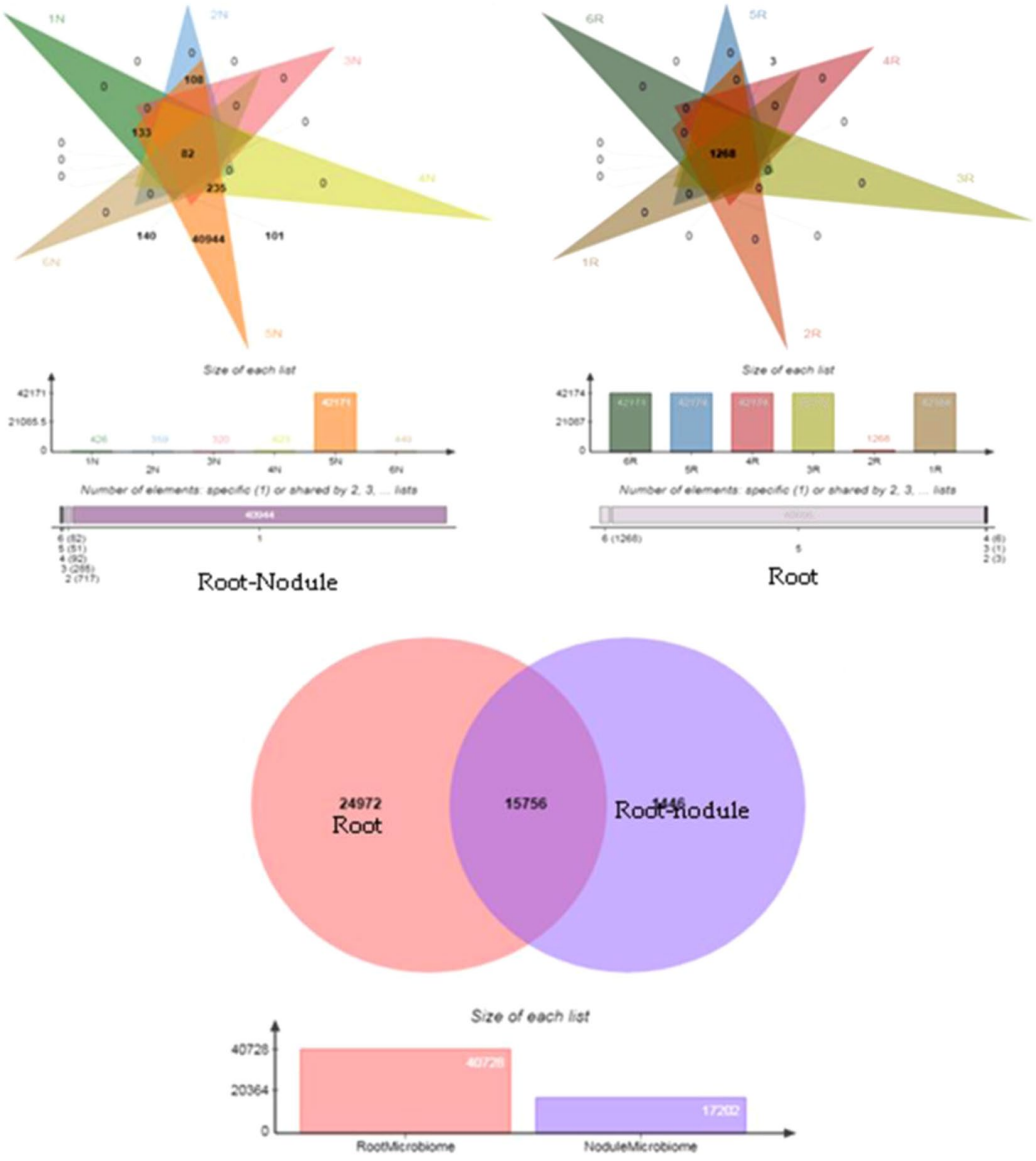
Venn diagram of root and nodule associated bacterial community of chickpea.

β-distance within the samples is lower compared to the distances between samples. Similarly, β-distance between nodule samples is lower than that of root samples for weighed, unweighed UniFrac as well as Bray-Curtis analysis (Fig. 4). Significance difference in the composition of bacterial community associated with root and nodule samples of non-*Bt* and *Bt*-chickpea was demonstrated by Principal Coordinate Analysis (PCoA). First two axes of PCoA plot represented 93.42% of the total variations (Fig. 5). Root and nodule microbiomes of tested samples were clustered into two with few exceptions like 2R (IPCa2) and 5N (IPCT10). Nodule associated bacterial community of IPCT10 is clustered separately from all other nodule samples of Non-*Bt* and *Bt*-chickpea (Fig. 5a). Similarly, root bacterial community of IPCa2 clustered away from remaining samples (Fig. 5b). It also indicated that the bacterial community structure of root is highly diverse in nature compared to that of nodule (Fig. 5c). Furthermore, ADONIS differences between bacterial community of root and root-nodule of tested chickpea lines were calculated (Table 4). The p-value of 0.01 indicates that the grouping of samples by individual is statistically significant. The R^2^ value indicates that approximately 26.8% of the variation in distances is explained by this grouping. High R^2^ value proved the expected variation among root and nodule samples are significant. Venn diagram depicted the number of common OTUs shared by tested samples. Only 82 OTUs were shared by all root nodule samples while the value was 1268 for root samples. Root microbiome possesses 24972 OTUs as unique while root nodule microbiome possesses 1446 unique bacterial OTUs. Root and nodule microbiome shared 15756 OTUs as common (Fig. 6).

**Table 4:** ADONIS differences between root and nodule samples:.

	Df	Sums of Sqs	Mean Sqs	F. Model	R2	pr(>F)
qiime.data$map[[opts$category]]	1	0.9952	0.99519	3.6699	0.26846	0.01
Residuals	10	2.7118	0.27118	—	0.73154	—
Total	11	3.707	—	—	1	—

## Discussion

Legumes derive 70% of their nitrogen requirement through SNF and contribute 50–70 million tonnes of nitrogen annually to agricultural ecosystem^20,21^. Potential of this complex process mainly depends on the host-phenotypic traits including nodule number and its biomass with the heritability exceeding 0.8^22,23^. *Bt*-transgenic crops have the potential to influence such natural biological processes in their agro-ecosystem owing to a) release of toxic *cry*-proteins into the soil via root exudates, and through decomposition of crop residues and b) site of *Bt*-transgene integration in host genome and further alterations in plant metabolism. Hence, symbiotic potential of any transgenics/cultivars of legumes should be ensured before their commercialization for maintaining ecosystem sustainability.

In the present study, integration of *Bt*-genes (*cry1Aabc* and *cry1Ac*) into host genome did not influence the signal transduction between *Bt*-chickpea and its microsymbiont and thus all *Bt*-chickpea events initiated nodulation similar to parent *cv* DCP 92-3. Significant variations observed on nodulation traits of *Bt*-chickpea events might be due to altered physiology of transgenic plants that further resulted in altered ability of the host to support nodule development during active vegetative stage. Earlier studied reported such reduction in nodule biomass up to 10 to 21% in transgenic RR-soybean without any yield penalty^18^. A number of QTLs including D1b, A1, C2, O, B1, H, B2, E, J, and I were identified for nodulation traits in soybean and integration of *Bt-*gene into any one of the or similar to above QTLs/genes involving C-metabolism can hamper SNF in legumes^22–26^.

*Bt*-transgenic crops can alter the bacterial community structure in agro-ecosystem including rhizosphere soil. Constitutive expression of some of the transgenic proteins due to cauliflower mosaic virus (CaMV) 35S RNA promoter, may act as toxins towards non-target endophytic bacterial community as well as rhizobacteria^27,28^. Earlier Prischl *et al*. showed that members of *Alphaproteobacteria*, *Actinobacteria* and *Gammaproteobacteria* were predominant in *Bt*-transgenic maize^29^. The available reports, however, are not consistent regarding the nature of interaction of transgenic crops with the native bacterial community. *Cry3Bb* and the *Cry1Ac* toxins persist for 21 and 56 days in soil microcosm and laboratory experiments, respectively^30–32^ and *Bt*-toxin is not retrieved from field soils for 3–6 consecutive years of *Bt*-cotton cultivation^33^. Hence, instead of assessing the changes in rhizobacterial community, we have analyzed the changes in endophytic bacterial community associated with root and root-nodules where the concentration of available *cry*-protein and its impact is relatively higher.

Endophytic colonization by bacteria is ubiquitous in nature and plants deprived of endophytes would be more vulnerable to biotic and abiotic stresses^34–37^. Endophytic bacterial isolates, belonging to *Enterobacteria*, *Pseudomonas*, and *Bacillus* were established in several surrogate hosts and improve their growth and yield^38^. In addition, they conferred stress tolerance to agricultural crops against drought, flood, heavy metal toxicity, and salinity by improving antioxidant defense mechanisms, maintaining ROS levels, producing ACC deaminase, altering the selectivity of K+, Na+ and Ca2+ and maintaining higher K+/Na+ ratio^39^. In the present investigation, endophytic bacterial community structure of root and root-nodules of *Bt*-chickpea were analyzed. Four major phyla detected in all tested *Bt*-chickpea tissues are *Proteobacteria* (0.35), *Cyanobacteria* (0.35), *Actinobacteria* (0.17), and *Firmicutes* (0.07). Similarly, Zhang *et al*. reported a greater relative abundance of *Proteobacteria* (14% to 68%), *Firmicutes* (26% to 41%), *Actinobacteria* (6 to 23%) and *Bacteroidetes* (1% to 21%) in plants struggling for existence in the extreme environments^38^.

Diversity and richness indices for bacterial community associated with roots of *Bt*-chickpea IPCa2 and root nodules of IPCT10 significantly differs from parent as well as other transgenic events. Gram positive bacterial Genera *Bacillus* and *Arthrobacter* were selectively eliminated from root colonization of IPCa2, while rests of the Non-Bt/Bt-chickpea events were colonized by the same along with *Streptomycetes*. Several species of *Streptomycetes* and *Bacillus cereus*, *B*. *subtilis*, and *B*. *amyloliquefaciens* were well-known for their potential on plant growth promotion and biological control of phyto-pathogens. Bacterial isolates belonging to *Arthrobacter* conferred stress tolerance and promoted plant growth under salt stress^40^. *Bt*-chickpea deprived of such beneficial endophytic colonization may suffer under stressed field environment. IPCa2 is the single transgenic event where the abundance of *Microcoleus, Methylobacillus, Thiomonas*, was increased in root tissues with reference to rest of the events. *Methylobacillus* of β-Proteobacteria detected only in IPCa2 utilizes methane, methanol and methylated amines^41,42^ and considered as one of the Earth’s most important carbon recyclers. *M. rhizosphaerae* was isolated from rhizosphere soil of red pepper (*Capsicum annuum* L. *cv* CO 1)^43^ and their interaction with agriculturally important plant is still to be explored.

Relative abundance of proteobacteria in root-nodules of chickpea ranges between 0.85–0.92 except IPCT10 with 0.42. *Mesorhizobium* is the most dominant bacteria in root-nodules with relative abundance ranging between 0.74–0.82. However, *Bt*-chickpea IPCT10 reported very low colonization of *Mesorhizobium* with 27.05% of total bacterial reads. Unlike other chickpea transgenic genotypes, root nodules of IPCT10 were also colonized by *Firmicutes*, *Actinobacteria* and *Cloroflexi* and thus its bacterial colonization pattern was similar to that of root tissues (Fig. 1). Though there was only slight reduction in the nodule biomass, poor symbiotic efficiency of these nodules were evidenced with significant reduction in nitrogen content of rhizosphere soil and chickpea shoot tissues at later stages (Table 2). The changes in the bacterial community structure of root nodules of IPCT10 may be due to poor symbiotic efficiency and vice versa. Root nodules of IPCT10 were detected with the presence of single species of *M. huakuii*. Interestingly, *M. amorphae* was detected only in root-nodules of IPCa2 that contributed 29.76% of its total *Mesorhizobium* reads. *M. septentrionale, M. camelthorni*, and *M. huakuii* are the other species detected in IPCa2.

A sharp decline in the percent reads classified from Genus to species level was observed in all tested samples (Fig. S2a). Large proportion reads of *Mesorhizobium* and *Rickettsia* were not classified at further taxonomic level (Fig. S2b), while the reads of remaining predominant bacterial Genera via. *Calothrix, Shinella*, and *Pseudaminobacter* were classified at their respective species level. Since the diversity of plant associated *Rickettsia* and chickpea associated *Mesorhizobium* in Indian soil is poorly studied^44–47^, most of the reads belongs to above Genera were not matched with existing species. At the same time, we need to consider the limitations on taxonomic resolution and accuracy of V3-V4 sequences to adequately perform species-level associative studies. In this study, read classification is performed using a high performance version of the RDP Naïve Bayes taxonomic classification algorithm built for most accurate species level classification that showed a genus-level concordance rate of 96% and a species-level concordance rate of 87.5%. Current result indirectly indicated that large diversity of *Mesorhizobium* in Indian soils is yet to be explored.

Several reports revealed that root-nodules are colonized to certain extend by non-rhizobial endophytic bacteria along with predominant host specific rhizobia. Incredibly diverse population of these endophytic bacteria neither induces root nodulation nor fixes nitrogen^48^. Metagenomic approach similar to our study revealed that the extent of soybean root-nodule colonization by *Bradyrhizobiaceae* was only 66% and the remaining bacteria belong to *Pseudomonadaceae* (19.8%), *Enterobacteriaceae* (11.8%), *and Paenibacillaceae* (5.3%) were considered as non-rhizobial endophytic bacteria^49^. Beneficial effect of such nodule-endophytes including *Micromonospora* on enhancing symbiotic efficiency without interfering with the host and its nodulating and nitrogen-fixing microbes was established^50^. Present study revealed an obvious decrease in root-nodule colonization of *Bt*-chickpea IPCT 10 by *Shinella, Pseudaminobacter, Burkholderia, Calothrix, and Mesorhizobium* compared to rest of the chickpea lines. *Shinella, Burkholderia and Pseudaminobacter* were detected only in root-nodule tissues and not found in root tissues of all tested chickpea events. *Shinella* exhibited a range of functional diversity including nitrogen fixation, belongs to *Rhizobiaceae* of *Alphaproteobacteria* and has been isolated from various environmental samples, like root nodules of herbal legume Kummerowia stipulacea, soils, and water^51^. *Pseudaminobacter*, a member belongs to *Phyllobacteriaceae* of *α*-*Proteobacteria*, has characteristic ability to utilize alkylamines and thus degrade widely used herbicide Atrazine (2-chloro-4-ethylamino-6-isopropylamino-1,3,5-triazine) in agricultural soils. However, its role on nodule metabolism is still unclear.

Species richness and Shannon diversity index was much higher in IPCT10 (1196, 2.327) than that of parent genotype (197, 0.823) respectively. High Shannon index of root nodules of event IPCT10 is similar to that of root tissues of parent genotype. It indicated that root nodules of transgenic event IPCT10 lost their unique properties, and hence colonized by soil microorganisms in a similar pattern to root tissues. Root and nodule microbiomes of tested samples were formed two separate clusters with few exceptions like 2R (IPCa2) and 5N (IPCT10) during PCoA analysis. Root associated bacterial community of IPCa2 is clustered separately from all other root samples of Non-*Bt* and *Bt-*transgenic line of chickpea. Similarly, nodule bacterial community of IPCT10 clustered away from remaining samples. The above analyses clearly indicated that the bacterial community structure of root is highly diverse in nature compared to that of nodule. Similarly, da Silva *et al*. reported that bacterial community structure of transgenic maize TC1507 tended to separate from those of MON810 and its near-isogenic parent^52^.

Apart from the impact of transgenic proteins, constitutive expression of neomycin phospho-transferase II (*npt*II) might also be responsible for changes in endophytic/rhizobacterial community of *Bt*-crops. Neomycin phospho-transferase II inactivates amino-glycoside antibiotics such as kanamycin and neomycin and thus disturbs the natural regulatory mechanism to maintain particular bacterial community structure in an ecosystem. Moreover, release of such antibiotic genes into in the soil by transgenic crops raised concerns about horizontal gene transfer (HGT) to rhizobacteria. High transfer frequency of plasmids with antibiotic resistance and acquisition of kanamycin resistant genes from transgenic plant DNA to the rhizosphere bacterium *Acinetobacter* sp. in soil microcosms have confirmed the possibility of bacterial natural transformation^53–56^. Apart from above environmental risks, reductions of nodule mass and BNF rates in transgenic plants and their long term cultivation in agricultural soil lead to increased scavenging of mineral N, depletion of organic matter reserves and lower the soil fertility especially in sandy soils with limited N availability^57^. With the above understanding, we emphasize the need of systematic risk assessment strategies for long term monitoring of SNF and soil health before and after releasing the transgenic crops.

## Materials and Methods

### Geographical location, description of field site and experiments

Confined field experiment to assess the effect of five *Bt*-transgenic events of chickpea (IPCa2, IPCa4, IPCT3, IPCT10, IPCT13) along with parent cultivar (cv. DCP 92-3) on biological nitrogen fixation and microbiome associated with root and root nodules was laid out under randomized complete block design (RCBD) at Plot No. 5/2, main research farm, ICAR-Indian Institute of Pulses Research, Kanpur, India with Geographical Co-ordinate: 26.49132°N, 80.27282°E. Soils in the experimental field are classified as inceptisol with nitrogen content of 281.9 kg/ha at 15 cm depth and 156.6 kg/ha at 30 cm depth. The basic properties of the soil were 46.98 g/kg organic matter, 0.51 g/kg total nitrogen, 11.73 mg/kg available phosphorus, 220.40 mg/kg available potassium, and 52.81 mg/kg alkali-hydrolyzed nitrogen.

### Plant/Soil sampling and analysis

*Bt*-transgenic lines were developed by employing *Agrobacterium tumefaciens* EHA105 mediated genetic transformation of chickpea *cv* DCP92-3^12^. For genetic transformation, the binary vector pBinAR originally derived from pBin19^58^, harboring either *Bt-cry1Aabc* or *Bt*-*cry1Ac* and neomycin phosphotransferase II (nptII) as the plant selectable marker gene (within T-DNA) was used. Domain shuffled *Bt-cry1Aabc* gene is expressed in IPCa2 and IPCa4 while plant codon optimized chimeric *Bt*-*cry1Ac* is expressed in IPCT3, IPCT10, and IPCT13 under the control of constitutive promoter CaMv35S. Plants were re-confirmed for the presence and expression of transgenes, using gene specific PCR and quantitative ELIZA as described^12^. The transgenic chickpea lines used in the study exhibited high trait efficacy against gram pod borer. Agronomic performance of the transgenic chickpea lines *vis-a-vis* control were conducted based on Guidelines for conduct of test for Distinctness, Uniformity and Stability (DUS) prescribed by PPV & FRA, Government of India. Twenty characters specific to chickpea genotype articulated in the Table of Characteristics (Section VII) of the Guidelines are being evaluated and recorded in all the transgenic lines *vis-a-vis* parental genotype, DCP92-3, at different stages (*viz*. seedling, pre-flowering, post-flowering and maturity). These characters are anthocyanin colouration, stem height at initiation of first flower, 50% flowering, growth habit, colour of foliage, leaflet size (middle of plant and leaf), leaf pattern, flower: number/peduncle, flower colour, flower: Stripes on standard, peduncle: length, plant height, pod size, number of seeds/pod, seed: colour, seed: Size (100 seed weight), seed shape, seed testa texture, seed ribbing and seed type. No significant differences for the above characteristics including average grain yield/plant were observed in the transgenic lines, as compared to control. Plant samples and associated rhizosphere soil in three replicates were collected at 63, 75, and 96 days after sowing (DAS) and subjected to further analysis along with bulk soil from the same field. Nitrogen content of plant samples as well as soil was determined by Kjeldahl method. The pH of the soil was measured in a soil: water ratio of 1:2.5.

### DNA extraction and next generation sequencing (NGS) of v3-v4 of 16S rDNA

Chickpea plants were uprooted from field soil carefully and transported in an ice-bucket to laboratory immediately. All samples were processed within few hours since their collection. Root and root nodules from three independent plants for each parent/transgenic lines were detached and then washed with sterile water followed by vortexed vigorously to remove adhered soil particle and dislodge surface microorganisms. Plant tissues were surface sterilized with 0.01% HgCl_2_ and 70% ethanol followed by several washings with sterile water to avoid residual effect. Root nodules with specific weight of 100–120 mg were selected and each ~1 g of root and root nodule tissues from parent/transgenic lines were homogenized with CTAB buffer in a Mortar and pestle and used for DNA extraction as described by Murray and Thompson^59^. Extracted DNA was tested for their quality and quantity through spectrophotometric analysis and stored at −20 °C. DNA from three replicated samples were pooled together and further subjected to the amplification of V3-V4 regions of 16S rDNA using Illuminia 16sRNA sequencing protocol with primer set Forward Primer = 5′ TCGTCGGCAGCGTCAGATGTGTATAAGAGACAGCCTACGGGNGGCWGCAG, and Reverse Primer = 5′ GTCTCGTGGGCTCGGAGATGTGTATAAGAGACAGGACTACHVGGGTATCTAATCC. Paired end read sequencing chemistry with MiSeq platform was performed at Sandor Life Sciences Pvt Ltd, Hyderabad, India and raw reads were submitted to NCBI SRA database under Bioproject Id: PRJNA578245.

### Bioinformatics

Paired end raw reads with 301 bp (Illumina MiSeq) were subjected to quality check/data processing by QIIME software^60^. Sequences with >97% identical are conventionally assumed to be derived from the same bacterial species. Open-reference OTU picking was used for analyzing current data. It starts with alignment to a reference database, and read does not match a known sequence is sent for *de novo* OTU picking. OTU information generated through OTU picking was utilized to estimate diversity within and between samples. Multiple rarefactions were generated and alpha diversity was measured on the rarefied OTU tables with tools available on QIIME. Heatmaps were drawn by using pheatmap, a package available in R-package^61^. The heat map depicts the relative abundance of each bacterial genus within each sample. The data is displayed in a grid where each row represents a genera and each column represents a sample. The colour and intensity of the boxes is used to represent relative values (Z-score values) for the bacterial genera. The value Zero in the color scale at the top right corner represents the mean. The value +2 represents two standard deviations above the mean and the value −2 represents two standard deviations below the mean. The green colour represents abundant genera and the blue represents less abundant genera. Principal Coordinate Analysis (PCoA) was carried out to measure of how similar or dissimilar the samples are, and is usually represented by a distance matrix which is then used to do PCoA. The result of this is an ordination plot of multiple dimensions, where each sample is a point and the distance between the points represents the similarity of those samples. The weighted UniFrac metric was used to determine the distance between samples PCoA showed similarity between normalized relative abundance of all samples. The PCoA is generated using Classical MDS on a Pearson covariance distance matrix generated from per-sample normalized classification abundance vectors. It measures differences in the distribution of taxonomic classifications between samples, up to a fixed taxonomic level.

### Statistics

Data are expressed as means ± SD. Significance was defined as P < 0.05. Venn diagrams were used to represent shared and unique rare OTUs among different sample groups. ADONIS: non-parametric statistical method that takes a QIIME distance matrix file such as a UniFrac distance matrix, a mapping file, and a category in the mapping files to determine sample grouping. It computes an R^2^ value (effect size) which shows the percentage of variation explained by the supplied mapping file category, as well as a p-value to determine the statistical significance. The p-value of 0.01 indicates that the grouping of samples by individual is statistically significant.

## Supplementary information

Supplementary Table S1

Supplementary Table S2

Supplementary Table S3

## Data Availability

Data generated and analyzed during this study are included in this published article
