# Supplementary material for: Symbiotic nitrogen fixation and endophytic bacterial community structure in Bt-transgenic chickpea (Cicer arietinum L)
Source: Sci Rep. 2020 Mar 25;10:5453. doi: 10.1038/s41598-020-62199-1 (PMC7096491; doi:10.1038/s41598-020-62199-1)
Supplement: Supplementary file 1 — Supplementary Table S1 [file 41598_2020_62199_MOESM1_ESM.doc]

Table S1: Phylum and genus level relative abundance of endophytic bacteria from Non-*Bt* and *Bt*-transgenic chickpea

| Phylum | Root-Nodules | | | | | | Root | | | | | | | | | | | |
| --- | --- | --- | --- | --- | --- | --- | --- | --- | --- | --- | --- | --- | --- | --- | --- | --- | --- | --- |
|  | DCP92-3 (1N) | IPCa2  (2N) | IPCa4  (3N) | IPCT3  (4N) | IPCT10  (5N) | IPCT13  (6N) | DCP92-3  (1R) | | IPCa2  (2R) | | IPCa4  (3R) | | IPCT3  (4R) | | IPCT10  (5R) | | IPCT13  (6R) | |
| *Proteobacteria* | 0.897833 | 0.903275 | 0.848215 | 0.887700 | 0.421010 | 0.916897 | 0.348115 | | 0.392143 | | 0.462585 | | 0.345523 | | 0.372117 | | 0.318628 | |
| *Cyanobacteria* | 0.098929 | 0.093755 | 0.149081 | 0.109637 | 0.023620 | 0.080484 | 0.348945 | | 0.574794 | | 0.128975 | | 0.18119 | | 0.162015 | | 0.222743 | |
| *Firmicutes* | 0.001059 | 0.001150 | 0.000786 | 0.000962 | 0.128767 | 0.000787 | 0.069550 | | 0.001571 | | 0.135080 | | 0.14834 | | 0.155723 | | 0.123195 | |
| *Actinobacteria* | 0.000987 | 0.000532 | 0.000754 | 0.000733 | 0.317728 | 0.000743 | 0.166949 | | 0.024753 | | 0.181345 | | 0.210168 | | 0.155426 | | 0.232482 | |
| *Bacteroidetes* | 0.000421 | 0.000567 | 0.000424 | 0.000321 | - | 0.000356 | - | | 0.003518 | | 0.011505 | | 0.011472 | | 0.022370 | | - | |
| *Chloroflexi* | 0 | - | - | - | 0.021505 | - | 0.009326 | | - | | 0.010942 | | 0.010434 | | 0.014234 | | 0.015961 | |
| *Verrucomicrobia* | - | 0 | 0 | - | - | - | - | | 0.000156 | | - | | - | | - | | - | |
| *Planctomycetes* | - | - | - | 0 | 0.011887 | 0 | 0.008420 | | - | | 0.010280 | | 0.012 | | 0.017507 | | 0.012302 | |
| *Gemmatimonadetes* | - | - | - | - | 0.011926 | - | 0.006366 | | - | | - | | - | | - | | 0.007708 | |
| Total Classified | 0.999278 | 0.999347 | 0.999324 | 0.999411 | 0.936443 | 0.999343 | 0.957671 | | 0.996935 | | 0.940712 | | 0.919126 | | 0.899392 | | 0.933020 | |
| Genus |  |  |  |  |  |  |  |  | |  | |  | |  | |  | |  |
| *Mesorhizobium* | 0.795197 | 0.820097 | 0.736463 | 0.791917 | 0.270449 | 0.801676 | 0.021100 | 0.008428 | | - | | - | | - | |  | |  |
| *Calothrix* | 0.097087 | 0.092398 | 0.146881 | 0.107798 | 0.022019 | 0.079148 | 0.342655 | 0.565474 | | 0.126159 | | 0.177125 | | 0.152927 | | 0.215086 | |  |
| *Rickettsia* | 0.065286 | 0.051634 | 0.075743 | 0.051370 | - | 0.068331 | 0.163180 | 0.321106 | | 0.063617 | | 0.091475 | | 0.085369 | | 0.13168 | |  |
| *Shinella* | 0.009954 | 0.007607 | 0.010468 | 0.016739 | - | 0.018931 | - | - | | -- | | - | | - | | - | |  |
| *Burkholderia* | 0.002684 | 0.002301 | 0.001525 | 0.001420 | - |  | - | - | | - | | - | | - | | - | |  |
| *Pseudaminobacter* | 0.002082 | 0.003056 | 0.003584 | 0.003972 | - | 0.005236 | - | - | | - | | - | | - | | - | |  |
| *Chelativorans* | 0.000734 | - | - | - | - | - | - | - | | - | | - | | - | | - | |  |
| *Methylobacillus* | - | 0.000704 | - | - | - | - | - | 0.004480 | | - | | - | | - | | - | |  |
| *Pseudomonas* | - | - | 0.000582 | - | - | - | - | - | | - | | - | | - | | - | |  |
| *Aminobacter* | - | - | - | 0.000720 | - | 0.000625 | - | - | | - | | - | | - | | - | |  |
| *Arthrobacter* | - | - | - | - | 0.037005 | - | 0.028207 | - | | 0.045908 | | 0.018017 | | - | | 0.027866 | |  |
| *Bacillus* | - | - | - | - | 0.029098 | - | 0.019936 | - | | 0.055109 | | 0.038277 | | 0.045217 | | 0.03157 | |  |
| *Streptomyces* | - | - | - | - | 0.018985 | - | - | 0.021336 | | - | | 0.027712 | | 0.012174 | | 0.017895 | |  |
| *Actinocatenispora* | - | - | - | - | 0.018978 | - | - | - | | - | | - | | - | | - | |  |
| *Saccharopolyspora* | - | - | - | - | 0.018278 | - | - | - | | - | | - | | -- | | 0.01152 | |  |
| *Rhodococcus* | - | - | - | - | - | - | 0.022755 | - | | - | | - | | - | | - | |  |
| *Ramlibacter* | - | - | - | - | - | - | 0.018084 | - | | 0.021948 | | - | | - | | - | |  |
| *Thiomonas* | - | - | - | - | - | - | - | 0.001798 | | - | | - | | - | | - | |  |
| *Microcoleus* | - | - | - | - | - | - | - | 0.001470 | | - | | - | | - | | - | |  |
| *Propionivibrio* | - | - | - | - | - | - | - | - | | 0.041549 | | 0.027448 | | - | | - | |  |
| *Janthinobacterium* | - | - | - | - | - | - | - | - | | 0.040727 | | - | | - | | - | |  |
| *Kaistobacter* | - | - | - | - | - | - | - | - | | - | | 0.016231 | | 0.027338 | | - | |  |
| *Sphingomonas* | - | - | - | - | - | - | - | - | | - | | - | | 0.013721 | | - | |  |
| *Ammoniphilus* | - | - | - | - | - | - | - | - | | - | | - | | 0.012026 | | - | |  |
| *Rubrobacter* | - | - | - | - | - | - | - | - | | - | | - | | - | | 0.014702 | |  |
| Total Classified | 0.973026 | 0.977798 | 0.975245 | 0.973936 | 0.414812 | 0.973948 | 0.615918 | 0.924092 | | 0.395017 | | 0.396284 | | 0.348773 | | 0.450319 | |  |
| Unclassified | 0.026974 | 0.022202 | 0.024755 | 0.026064 | 0.585188 | 0.026052 | 0.384082 | 0.075908 | | 0.604983 | | 0.603716 | | 0.651227 | | 0.549681 | |  |
