# Supplementary material for: Symbiotic nitrogen fixation and endophytic bacterial community structure in Bt-transgenic chickpea (Cicer arietinum L)
Source: Sci Rep. 2020 Mar 25;10:5453. doi: 10.1038/s41598-020-62199-1 (PMC7096491; doi:10.1038/s41598-020-62199-1)
Supplement: Supplementary file 2 — Supplementary Table S2 [file 41598_2020_62199_MOESM2_ESM.doc]

Table S2: Diversity of *Mesorhizobium* in root nodules of Non-Bt and *Bt*-transgenic chickpea

| **Plant type** | **Total Reads** | ***Mesorhizobium*** | | **Known *Mesorhizobium* species** | | |
| --- | --- | --- | --- | --- | --- | --- |
|  |  | Reads | % | *Mesorhizobium* sp. | Number of Reads | % |
| Chickpea *cv* DCP92-3 | 83080 | 66065 | 79.52 | *M. huakuii*  *M. septentrionale*  *M. camelthorni*  *M. opportunistum*  *TOTAL* | 3548  3464  298  42  7352 | 11.13% |
| IPCa2 | 58237 | 47760 | 82.01 | *M. a*morphae  *M. septentrionale*  *M. camelthorni*  *M. huakuii*  *TOTAL* | 14215  2380  207  178  16992 | 35.56% |
| IPCa4 | 63623 | 46856 | 73.65 | *M. septentrionale*  *M. camelthorni*  *TOTAL* | 2522  299  2821 | 6.02% |
| IPCT3 | 152814 | 121016 | 79.19 | *M. septentrionale*  *M. huakuii*  *M. camelthorni*  *M. opportunistum*  *TOTAL* | 2817  625  392  109  3943 | 3.26% |
| IPCT10 | 155545 | 42067 | 27.05 | *M. huakuii* | 1365 | 3.25% |
| IPCT13 | 92813 | 74406 | 80.17 | *M. huakuii*  *M. septentrionale*  *M. camelthorni*  *M. opportunistum*  *TOTAL* | 2303  1563  158  114  4138 | 5.56% |
